# Weakly supervised label propagation algorithm classifies lung cancer imaging subtypes

**DOI:** 10.1038/s41598-023-32301-4

**Published:** 2023-03-30

**Authors:** Xueting Ren, Liye Jia, Zijuan Zhao, Yan Qiang, Wei Wu, Peng Han, Juanjuan Zhao, Jingyu Sun

**Affiliations:** 1 College of Information and Computer, Taiyuan University of Technology, Taiyuan, Shanxi China; 2 Department of Clinical Laboratory, Affiliated People’s Hospital of Shanxi Medical University, Shanxi Provincial People’s Hospital, Taiyuan, Shanxi China; 3 North Automatic Control Technology Institute, Taiyuan, Shanxi China

**Keywords:** Cancer imaging, Lung cancer, Computational science, Computer science

## Abstract

Aiming at the problems of long time, high cost, invasive sampling damage, and easy emergence of drug resistance in lung cancer gene detection, a reliable and non-invasive prognostic method is proposed. Under the guidance of weakly supervised learning, deep metric learning and graph clustering methods are used to learn higher-level abstract features in CT imaging features. The unlabeled data is dynamically updated through the k-nearest label update strategy, and the unlabeled data is transformed into weak label data and continue to update the process of strong label data to optimize the clustering results and establish a classification model for predicting new subtypes of lung cancer imaging. Five imaging subtypes are confirmed on the lung cancer dataset containing CT, clinical and genetic information downloaded from the TCIA lung cancer database. The successful establishment of the new model has a significant accuracy rate for subtype classification (ACC = 0.9793), and the use of CT sequence images, gene expression, DNA methylation and gene mutation data from the cooperative hospital in Shanxi Province proves the biomedical value of this method. The proposed method also can comprehensively evaluate intratumoral heterogeneity based on the correlation between the final lung CT imaging features and specific molecular subtypes.

## Introduction

The average 5-year survival rate of patients with advanced lung cancer (stage II, III, IV) is less than 5%, and the median survival period of patients with advanced lung cancer is 6–8 months^1^. Therefore, using a new treatment method to improve the survival rate of lung cancer patients and even achieve cure has become a major problem in the medical field. However, the emergence of targeted therapy has raised the hope of rebirth for patients with advanced lung cancer^2,3^, and it can also provide clinicians with a more systematic and comprehensive treatment direction. However, the current clinical implementation of targeted therapy has the problems of long time for gene detection, high cost and large damage of invasive sampling. For patients with advanced stage, it is of great significance to find a method that can effectively replace the effect of gene detection. It does not need to conduct puncture biopsy for patients, nor spend time waiting for the results of gene sequencing to find the target. Previously, Nature^4^ published an article on image genomics, which confirmed the correlation between prostate cancer genes and imaging, and revealed the important role of its correlation in cancer diagnosis and prognosis. If the mapping correlation between the image and the pathogenic gene in the imaging lesions can be used to directly predict the pathogenic mutation gene from the image and provide a new scheme for targeted therapy, it can not only buy time for patients, but also reduce the waste of medical resources. At the same time, the image can be used to verify the relevant pathogenic gene detected by the gene, further improve the accuracy of the detection results, and achieve the goal of precise drug recommendation.

In the multi-modal data of medical images, Computed Tomography (CT)^5,6^ images obtained by computer tomography can not only detect the density, gray scale and other information of tumor tissues, find the subtle differences between tissues, and evaluate the characteristics of tumor tissues non invasively, but also have low cost and high efficiency. It is easier to obtain medical images of small samples, and has become a routine clinical practice for tumor diagnosis, staging and treatment guidance^7^. The National Lung Screening Test (NLST) has proved that lung cancer screening through CT imaging can improve cancer prognosis^8^. Therefore, using CT images to classify the imaging subtypes of lung cancer can further find the correlation between imaging subtypes and gene mutation targets. There are many previous literatures that have studied the relationship between medical imaging features and molecular subtypes. However, because the identification of molecular subtypes is limited by the requirements of invasive biopsy, researchers try to determine whether medical imaging features can help to identify the molecular subtypes of cancer. For example, Mazurowski et al.^9^ extracted 23 imaging features from quantitative magnetic resonance (MR) images of breast cancer, determined molecular subtypes on the basis of genome analysis, and evaluated the correlation between imaging features and molecular subtypes using logistic regression and likelihood ratio test. Wu et al.^10^ extracted imaging features and tumor molecular subtypes of breast cancer and conducted univariate and multivariate logistic regression analysis. It was found that various molecular subtypes showed strong correlation with corresponding imaging features.

Based on this, this paper attempts to cluster imaging subtypes by identifying the correlation between lung CT image features and specific molecular subtypes. At present, there has been some research on cancer subtype classification methods based on image features. Wu et al.^11^ identified new breast cancer subtypes by extracting quantitative imaging phenotypes of tumors and their surrounding parenchyma, constructed an image subtype classifier based on gene expression, and tested their prognostic significance using samples with gene expression data but no image data. Itakura et al.^12^ distinguished GBM subtypes only through MR imaging features, extracted quantitative image features of the shape, texture and edge sharpness of each lesion, and identified three new GBM subgroups. In this study, we try to establish subtypes of lung cancer biomarkers based on CT images, so that images can eventually replace intensive molecular analysis, that is, image processing based methods can be used to avoid the risk of biopsy, and more comprehensively evaluate the heterogeneity within tumors^2^.

Cluster analysis is one of the most simple and important methods for cancer subtype classification at present. Most of the existing clustering methods use unsupervised learning to classify unlabeled data^13^, which can be roughly divided into three categories: (1) feature learning clustering. More discriminant features can be obtained by combining data dimension reduction technology or subspace learning technology; (2) Measure learning clustering. By learning an appropriate distance metric for training data, similar samples are more clustered and different samples are more separated; (3) Graph clustering. By expressing the data as a graph, the clustering problem is transformed into a graph partition problem, and the data is divided into different classes according to the paired similarity of the data. On this basis, some deep learning based methods^14^ are also used to solve clustering problems. How to extract useful features and learn appropriate metrics is a challenging task for high-dimensional data without any supervision information. Therefore, some supervised clustering algorithms^15^ have been proposed to improve the clustering results. However, in most cases, it is difficult to obtain a large number of labeled data from medical image data. For this reason, this paper proposes a Weak Supervision Deep Metric learning and Graph Clustering (WS-DMGC) algorithm, which makes full use of the advantages of label propagation strategy, and converts a large number of unlabeled data into weak label data first, and then into strong label data to improve the clustering performance, and automatically and accurately capture new subtypes of lung cancer with the impact of genetic characteristics when only imaging data is used.

The contributions of this paper are summarized as follows:

1. We proposed WS-DMGC, a new deep metric learning and graph clustering method, which uses the triplet loss as the loss function of the metric learning network. At the same time, we improve the selection strategy on the triplet, which makes the model more efficient and accelerates the convergence speed of the model.
2. The paper is specially designed to effectively use a small number of labeled samples to predict the new subtypes of lung cancer imaging, and solve the problem that it is difficult to obtain the labeled samples of clinical medicine imaging, which affects the classification performance of the weak supervision model.
3. A large number of lung cancer CT image datasets are characterized in an end-to-end manner. A metric space based on the triplet CNN model is designed, which jointly retains the ability to distinguish the lung cancer imaging subtypes of labeled and unlabeled samples.
4. We have carried out extensive experimental evaluation on two kinds of lung cancer image datasets, and compared the performance of the proposed method with different state-of-the-art methods. The experimental results verify the effectiveness and robustness of the method.

The content of the remaining sections of this article is arranged as follows. “Related work” introduces the relevant methods mentioned in this article. “Materials and methods” introduces the algorithm proposed in this paper. The experiment and parameter design are given in “Results”. “Discussions” discusses the experimental results, and uses survival analysis and gene analysis to predict and evaluate the imaging subtypes of different lung cancer patients. “Conclusion” concludes.

## Related work

### Deep metric learning based on weakly supervised clustering

Previous studies have shown that the clustering method based on weak supervision can be used to improve the performance of the training model^16^. Unlike unsupervised learning, weakly supervised learning uses a small number of labeled samples and a large number of unlabeled samples for training. For example, Guan et al.^17^ proposed a feature space learning model based on a weakly supervised framework to better represent and learn the feature space. Laine et al.^18^ proposed a time ensemble model based on weakly supervised learning. Li et al.^19^ proposed a local density model to measure the similarity between k-adjacent vertices. Kang et al.^20^ combined multi-core learning and weak supervision techniques to solve the clustering problem.

**Figure 1:**
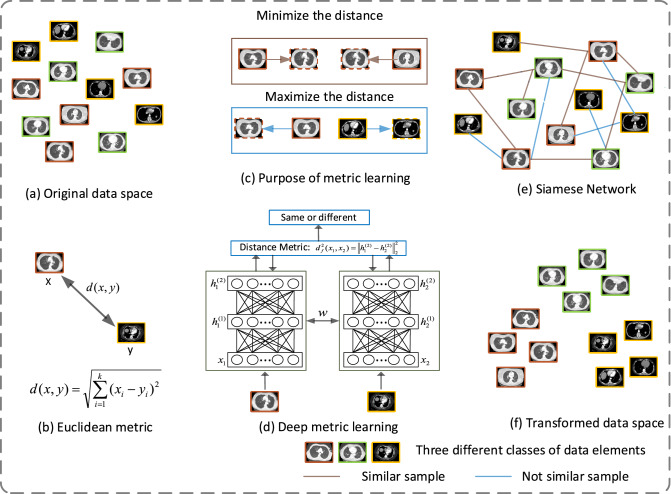
Block diagram of deep metric learning based on weakly supervised clustering (**a**) original data space (**b**) Euclidean metric (**c**) the purpose of metric learning (**d**) deep metric learning (**e**) Siamese network (**f**) transformed data space.

Compared with the above traditional clustering methods based on weak supervision, the method of combining weak supervision and deep learning can learn distinctive features more comprehensively and improve the accuracy of clustering. For example, Mai et al.^21^ proposed a weakly supervised deep fuzzy C-means clustering model. Ren et al.^22^ proposed a semi-supervised deep embedding clustering model. Shukla et al.^23^ designed a ClusterNet model to promote the clustering effect through paired semantic constraints. Caron et al.^24^ proposed a clustering method, DeepCluster, which combines the two tasks of clustering and classification. The features obtained by the network are directly used for clustering, and the cluster labels are used as supervision to update the weights of the network, suitable for label scarcity areas. Therefore, we add deep metric learning^25,26^ to optimize the clustering performance in this paper, so as to obtain better clustering results. So far, the deep metric learning method has been applied to some images processing problems^16,19^. In this paper, we propose a weakly supervised deep metric learning network to reduce the distance between the same type of samples and increase the distance between different types of samples. Figure 1a–f shows the block diagram of deep metric learning based on weakly supervised clustering proposed in this paper.

Traditional Mahalanobis distance metric learning is to find matrix $M\in {R}^{d\times d}$ from the training set *X* to calculate the Mahalanobis distance between two samples ${x}_{i}$ and ${x}_{j}$. The calculation method can be obtained by Eq. (1):

1 $$\begin{aligned} d_{M} (x_{i} ,x_{j} ) & = \sqrt {\left( {x_{i} - x_{j} } \right)^{T} M\left( {x_{i} - x_{j} } \right)} \\ & = \sqrt {\left( {x_{i} - x_{j} } \right)^{T} W^{T} W\left( {x_{i} - x_{j} } \right)} \\ & = \left\| {W_{{x_{i} }} - W_{{x_{J} }} } \right\|_{2} \\ \end{aligned}$$

Since *M* is a symmetric positive semi-definite matrix, it can be decomposed into ${M=W}^{T}W$, of which $W\in {R}^{p\times d}$*, p* < *d*. According to Eq. (1), it can be seen that the traditional Mahalanobis distance metric learning is to project each sample ${x}_{i}$ into a low-dimensional subspace by finding a linear transformation (because *p* < *d*), and the Euclidean distance between the samples after projection is the Mahalanobis distance in the original space. Because the linear transformation used in the traditional method cannot capture the nonlinear manifold that lung cancer images rely on, in order to solve the limitation of the traditional method, this paper projects the sample into a high-dimensional feature space and measures the distance in the high-dimensional space.

**Figure 2:**
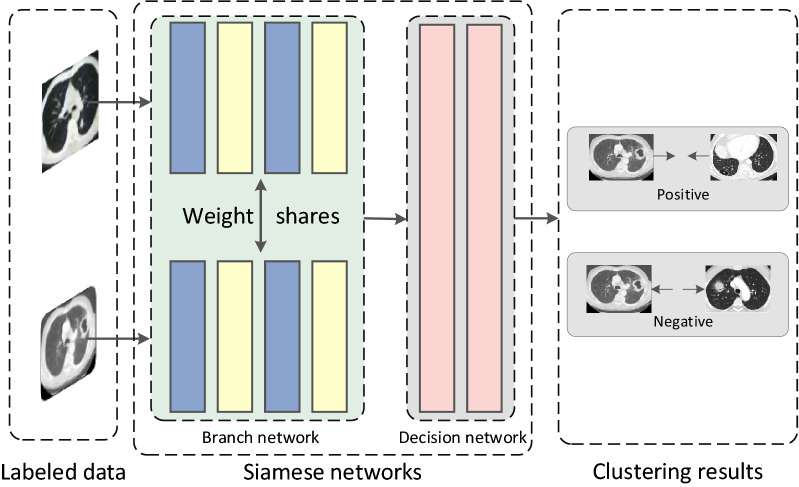
Weakly supervised classification model based on deep metric learning (Color code used: blue = Conv + ReLU, yellow = max pooling, pink = fully connected layer, ReLU exists between fully connected layers as well, the two branches in each stream are shared in this case).

As shown in Fig. 2, the traditional Siamese CNN-based weakly supervised deep metric learning network first inputs the labeled sample pairs of lung cancer CT images into the Siamese neural network, there are two branches in the network that share the same architecture and weights. Each branch takes one of the CT image pairs as input, then passes through a series of convolutional layers, ReLU layers and maximum pooling layers, and finally uses linear fully connected layers and ReLU layers to form a top-level network, and outputs identifiable features. The branch of the Siamese network can be regarded as a descriptor calculation module, and the top-level network can be regarded as a similar function. For the task of matching two sets of images during the test, first use the branch to independently calculate the descriptors, and match with the top-level network to train the deep metric network, and then use the trained metric learning network to encode all labeled data and unlabeled data Feature representation, classify unlabeled data according to coding features, and use the classification result as the label of unlabeled data.

In the feature learning process of the traditional Siamese neural network, the contrast loss is used as the objective function of the network. This loss function can effectively deal with the relationship of the paired data in the Siamese neural network and the matching degree of the samples. The calculation method of the loss function is shown in Eq. (2):

$$L(W,(Y,{X}_{1},{X}_{2}))=\frac{1}{2N}\sum_{n=1}^{N}Y{D}_{W}^{2}+(1-Y)max{(m-{D}_{W},0)}^{2}$$

2 $${D}_{W}\left({X}_{1},{X}_{2}\right)={\Vert {X}_{1}-{X}_{2}\Vert }_{2}={\left(\sum_{i=1}^{P}{\left({x}_{1}^{i}-{x}_{2}^{i}\right)}^{2}\right)}^\frac{1}{2}.$$

Among them, ${D}_{W}$ represents the Euclidean distance between the two sample features ${x}_{1}$ and ${x}_{2}$, ${x}_{1}$ and ${x}_{2}$ respectively represent the features of the input sample pair extracted by the deep metric learning network, *P* represents the feature dimension of the sample, and *Y* is whether the two samples match (If *Y* is 1, it means that the input pairs are from the same class; if *Y* is 0, it means they are from different classes), *m* is the set threshold, and *N* is the number of samples.

In order to extract more discriminative features to optimize the clustering model, this paper uses the triplet neural network and uses the triplet loss as the loss function of the deep metric learning. In the next section, the k-nearest neighbor label update strategy is proposed, which dynamically converts unlabeled data into labeled data, and makes full use of the contribution of unlabeled data.

### K-nearest neighbors label updating strategy

This paper uses the k-nearest neighbors label update strategy^19^ to convert unlabeled data into labeled data. All data is divided into *n* type clusters, and each cluster contains a certain amount of labeled data and a large amount of unlabeled data. In order to make full use of the features of unlabeled data, Each time $k \times n$ new unlabeled data are added to the labeled data set. Algorithm 1 is the K-nearest neighbor label update strategy proposed in this paper.
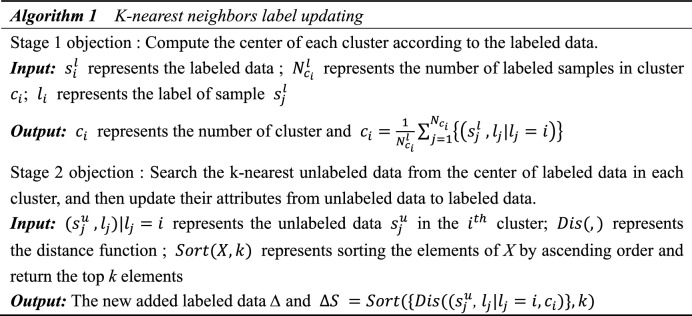


**Figure 3:**
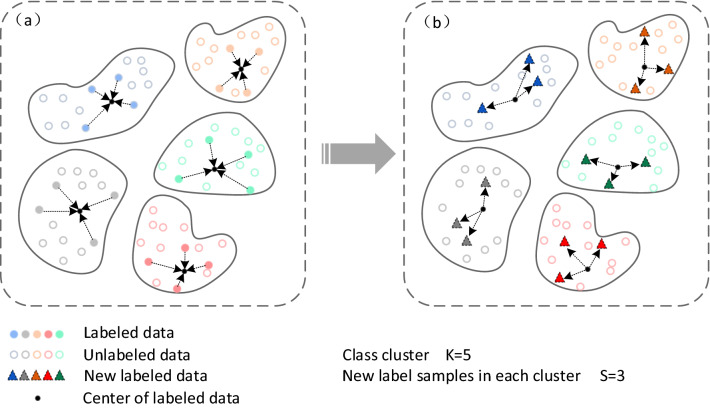
K-nearest neighbors (KNN) label updating.

As shown in Fig. 3 (labeled samples and newly labeled samples are distinguished by different colors and shades of graphics), all samples are divided into five clusters (K = 5), each cluster contains a small number of labeled samples and a large number of unlabeled samples, in which the solid points in each cluster represent labeled samples, and the hollow points represent unlabeled samples. After finding the cluster center of the labeled sample in each cluster (as shown in Fig. 3a), three new labeled samples will be generated (S = 3, as shown in Fig. 3b). These three samples are the unlabeled samples closest to the labeled sample center. As the number of labeled samples increases, the tag propagation strategy proposed in this paper can learn more identification features, thus further improving the accuracy of clustering.

The label propagation strategy has been widely used in many fields, but few studies have applied the label propagation strategy to the classification of lung cancer CT imaging subtypes. The k-nearest neighbor label update strategy is a form of label propagation, which is used in our work. The method is used to generate pseudo-labeled data, that is, to convert unlabeled data into strong-labeled data. In this case, the network is pre-trained with labeled data, and then the model is continuously updated with unlabeled data. And finally the optimal clustering result is obtained.

## Materials and methods

Due to the difficulty of obtaining medical image data annotation, existing methods can not make full use of unlabeled data to optimize and guide the clustering process. For this reason, this paper proposes a Weakly Supervised Deep Metric learning and Graph Clustering (WS-DMGC) algorithm, which uses the deep metric learning model to obtain the distinguishing features in unlabeled data, and uses graph based k-neighbor label propagation strategy to optimize clustering. This propagation strategy uses a trained classifier to classify the data carefully annotated by clinicians to provide high-quality pseudo tags. By fully learning the characteristics of large-scale unlabeled data, and using the tag propagation strategy to infer the pseudo tags of unlabeled data, the unlabeled data is dynamically converted to weak label data, and then the weak label data is converted to strong label data, so as to improve the clustering performance of the network.

**Figure 4:**
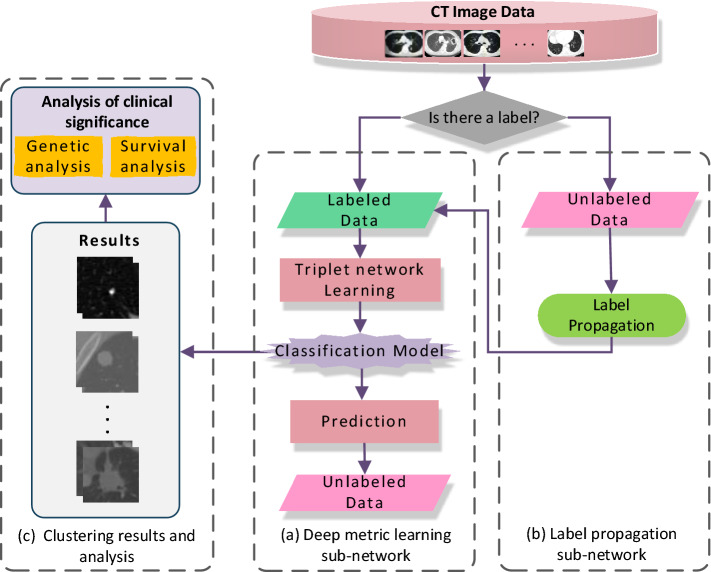
Block diagram of the proposed of lung cancer imaging subtype classification based on label propagation model (**a**) Deep metric learning sub-network (**b**) Label propagation sub-network (**c**) Clustering results and analysis.

As shown in Fig. 4, the block diagram of lung cancer imaging subtype classification proposed in this paper. Figure 4a is a deep metric learning subnetwork that uses a triplet neural network to extract discriminative features to reduce clustering error, and uses the triplet loss as the loss function of the deep metric learning network. Triplet neural networks consist of three samples from the same feedforward network with shared parameters. For training, we use an online triplet mining strategy (selecting positive/negative samples from a small batch) to generate roughly aligned matched/mismatched lung cancer CT imaging subtype triples, so as to speed up the model convergence, and train the entire network through a loss function. Under the triplet loss function, positive samples will be pulled towards the anchor and negative samples will be pushed away from the anchor. By decreasing the distance between positive samples and increasing the distance between negative samples, all labeled data can be clustered in the learned feature space. Figure 4b is a label propagation subnetwork. The k-neighbor label update strategy is used to combine the classification results of the labeled samples with the classification results of the unlabeled samples obtained by the improved graph clustering algorithm in this paper, and the increment of the labeled samples is continuously updated to optimize the clustering model, which enhances the ability of the deep metric learning network to obtain the optimal clustering results. Figure 4c carries out genetic analysis and survival analysis of the clustering results in this paper to clarify the clinical significance of lung cancer imaging subtypes, and combines the key gene expression characteristics to realize the automatic classification of lung cancer lesion image features.

Specifically, the proposed method will iterate between two steps. Firstly, the labeled samples were input, the triple CNN^27^ was used as the deep metric learning model, and the triple loss function was used to replace the contrastive loss function in the traditional Siamese network to train the network. Then the network trained in the previous step is used to input the unlabeled samples, and the weak labels of the unlabeled samples are obtained. Finally, we use a label propagation strategy to infer strong labels for unlabeled samples as well as subtype categories for each sample. All samples are trained using the determined weights. In addition, the more labeled samples available, the better our clustering effect, and the more significant the advantage of the proposed method.

**Figure 5:**
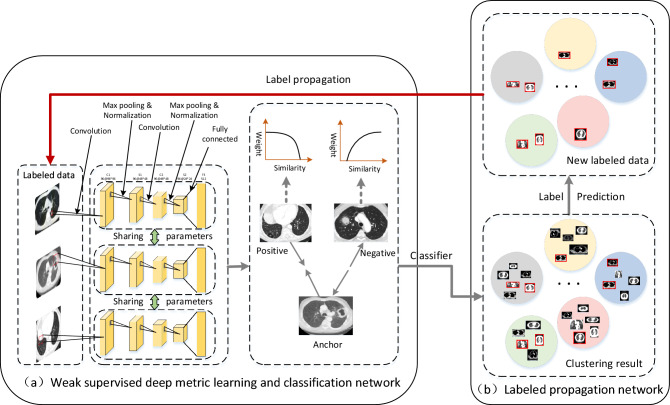
The framework of the proposed WS-DMGC. The framework consists of two subnetworks: (**a**) a feature extraction sub-network by Triplet CNNs; (**b**) a label propagation sub-network by graph clustering algorithm, circles in different colors indicate the clustering results for each category.

As shown in Fig. 5, the WS-DMGC network consists of two parts, namely the weakly supervised deep metric learning classification network in Fig. 5a and the label propagation network in Fig. 5b. Different from the other deep metric learning network in the literature, for training, this paper uses an online triplet mining method (select positive/negative samples from a small batch of samples) to generate roughly aligned matched/unmatched triplets of lung cancer CT images. The advantage of our method is that it has higher representation efficiency. The most important part is the end-to-end learning of the whole system. For this reason, we use triple loss to directly reflect the goal we want to achieve in the recognition and clustering of lung cancer imaging subtypes.The specific implementation steps of WS-DMGC network are as follows:

First, the triplet loss embedding is represented by $f(x)\in {R}^{d}$, which the image $X$ is embedded in a d-dimensional Euclidean space. We strive to find an embedding $f(x)$, from image $X$ to feature space ${R}^{d}$, such that the square distance between all CT image pairs of the same class is small, while the square distance between CT image pairs of different classes is large. Therefore, we would like to see Eq. (3)

3 $${\Vert {x}_{i}^{a}-{x}_{i}^{p}\Vert }_{2}^{2}+\alpha <{\Vert {x}_{i}^{a}-{x}_{i}^{n}\Vert }_{2}^{2}, \quad \forall \left({x}_{i}^{a},{x}_{i}^{p},{x}_{i}^{n}\right)\in \tau ,$$

where $\mathrm{\alpha }$ is the boundary enforced between positive and negative sample pairs. $\tau$ is the set of all possible triples in the training set. The minimized loss is shown in Eq. (4)

4 $${L}_{M}=\sum_{i=1}^{N}{[\Vert f({x}_{i}^{a})-f({x}_{i}^{p})\Vert }_{2}^{2}-{\Vert f({x}_{i}^{a})-f({x}_{i}^{n})\Vert }_{2}^{2}+\alpha ].$$

$f({x}_{i}^{a})$, $f({x}_{i}^{p})$ and $f({x}_{i}^{n})$ represent the characteristics of anchor points, positive samples and negative samples, respectively. $\alpha$ is the minimum boundary value of ${\Vert f({x}_{i}^{a})-f({x}_{i}^{p})\Vert }_{2}^{2}$ and ${\Vert f({x}_{i}^{a})-f({x}_{i}^{n})\Vert }_{2}^{2}$. Generating all possible triples will lead to many easily satisfied triples (that is, satisfying the constraints in Eq. (3)). These triples are not helpful for training and result in slower convergence because they will still pass through the network. To ensure fast convergence, it is crucial to choose a triplet that violates the triplet constraint (Eq. 3), which means that, given ${x}_{i}^{a}$, we choose a ${x}_{i}^{p}$ such that ${argmax}_{{x}_{i}^{p}}{\Vert {f(x}_{i}^{a})-f({x}_{i}^{p})\Vert }_{2}^{2}$, and again for ${x}_{i}^{n}$, such that ${argmin}_{{x}_{i}^{n}}{\Vert {f(x}_{i}^{a})-f({x}_{i}^{n})\Vert }_{2}^{2}.$

It is not feasible to calculate $argmin$ and $argmax$ through the whole training set. In addition, it may lead to poor training due to mislabeling and poor image quality. Therefore, this paper adopts the strategy of online generation of triples to calculate $argmin$ and $argmax$ only in small batches. To ensure that the anchor-to-positive sample distance is meaningful, we sample the training data such that each category occurs in each mini batch and, in addition, randomly sampled negative samples are added to each mini batch. This strategy of selecting triples improves the rapid convergence of the model. The gradients of the three samples are $\frac{\partial {L}_{M}}{\partial f({x}_{i}^{a})}$ *, *$\frac{\partial {L}_{M}}{\partial f({x}_{i}^{p})}$*, *$\frac{\partial {L}_{M}}{\partial f({x}_{i}^{n})}$*,* respectively.

In addition, in order to train the triple neural network and the classification network simultaneously, the overall loss function is shown in Eq. (5):

5 $$min \; L={L}_{M}+{\lambda }_{1}{L}_{C}+{\lambda }_{2}{\Vert W\Vert }_{F}^{2}$$

$${L}_{C}=-\sum_{f(x)}p(f(x))log \; q(f(x))$$

Among them, the determination of the ${\lambda }_{1}$ and ${\lambda }_{2}$ parameter values determines the accuracy of clustering. The impact of parameter evaluation on the clustering effect will be discussed in detail in this paper. ${\Vert W\Vert }_{F}^{2}$ is to prevent overfitting symbols, and ${L}_{M}$ and ${L}_{C}$ are respectively the metric learning loss and classification loss, $q(f(x))$ is the actual output of the classification network.

Second, encode the labeled samples and unlabeled samples. Assumptions ${S}_{l}=\{({s}_{{l}_{i}}, {l}_{{l}_{i}})|i=\mathrm{1,2},...,{N}_{l}\}$ and ${S}_{u}=\{({s}_{{u}_{i}}, {l}_{{l}_{i}})|i=\mathrm{1,2},...,{N}_{l}\}$ respectively represent the initial labeled samples and unlabeled samples, where ${N}_{l}$ represents the number of labeled samples and ${N}_{u}$ represents the number of unlabeled samples. ${l}_{{l}_{i}}$∈{1, 2, …, n}, where n represents the number of cluster categories.Use ${S}_{l}^{^{\prime}}=\left\{{s}_{{l}_{i}}^{^{\prime}}|i=\mathrm{1,2},\cdots ,{N}_{l}\right\}$ and ${S}_{u}^{^{\prime}}=\left\{{s}_{{u}_{i}}^{^{\prime}}|i=\mathrm{1,2},\cdots ,{N}_{u}\right\}$ to represent ${S}_{l}$ and ${S}_{u}$ outputs.

Finally, according to the classification network to label the unlabeled samples, ${S}_{u}$ can be expressed as ${S}_{u}$ = $\left\{{s}_{{u}_{i}},{l}_{{u}_{i}}^{1}| i=\mathrm{1,2},...,{N}_{u}\right\}$, where ${l}_{{u}_{i}}^{1}$ is the classification label of ${s}_{{u}_{i}}$.

The work of the above three steps allows us to obtain the weak label of the unlabeled sample, in which the triple loss is further added on the basis of the contrast loss, because the pseudo label we obtain is directly inferred by the label propagation strategy rather than the network prediction. In this case, the network is pre-trained, and the previously proposed graph clustering algorithm is a form of label propagation. This article will use an improved graph clustering algorithm to achieve strong labels for unlabeled samples in the process of iterative label propagation. This formula is also applicable to other fields. The specific steps are as follows:

First, calculate the similarity matrix *W* according to Eq. (6):

6 $${\omega }_{ij}=\left\{\begin{array}{c}exp \frac{{-\Vert {x}_{i}-{x}_{j}\Vert }^{2}}{2{\sigma }^{2}}\left\{{x}_{i},{x}_{j}\right\} \in {S}_{u}^{^{\prime}}\\ 1 \left\{{x}_{i},{x}_{j}\right\}\in {S}_{l}^{^{\prime}}\wedge \left\{{l}_{{x}_{i}}={l}_{{x}_{j}}\right\}\\ 0 \left\{{x}_{i},{x}_{y}\right\}\in {S}_{l}^{^{\prime}}\wedge \left\{{l}_{{x}_{i}}\ne {l}_{{x}_{j}}\right\}.\end{array}\right.$$

Among them, *σ* represents the neighborhood width of the sample point, the larger the *σ* is, the greater the similarity between the sample points.

Second, use Eq. (7) to calculate the degree matrix *D*:

7 $${d}_{i}=\sum_{i=1}^{n}{w}_{ij}.$$

The corresponding Laplacian matrix is obtained, as shown in Eq. (8):

8 $$L =D-W$$

Finally, use top $k$ feature vectors ${u}_{1}$*,*${u}_{2}$* …,* ${u}_{k}$ in $L$ to form a new matrix $U$ and obtain the clustering results. Mark ${S}_{u}^{^{\prime}}$ according to the clustering result, which is recorded as ${S}_{u}^{^{\prime}}=\left\{{S}_{{u}_{i}}^{^{\prime}},{L}_{{u}_{i}}^{2}|i=\mathrm{1,2},\cdots ,{N}_{2}\right\}$,where ${L}_{{u}_{i}}^{2}$ is the cluster label of ${S}_{{u}_{i}}^{^{\prime}}$. When the classification label ${L}_{u}^{1}$ and the cluster label ${L}_{u}^{2}$ of the unlabeled sample ${S}_{u}$ are obtained at the same time, the label propagation strategy can be realized. Assumption $\Delta S$ represents the strong label data obtained through a series of iterative updates, can be obtained by Eq. (9):

9 $$\Delta S=\left\{{S}_{{u}_{i}}|{(l}_{{u}_{i}}^{1}={l}_{{u}_{i}}^{2})\right\}.$$

Update ${\mathrm{S}}_{\mathrm{l}}$ and ${\mathrm{S}}_{\mathrm{u}}$ according to Eq. (10) until all unlabeled data are converted to labeled data.

$${S}_{l}={S}_{l}+\Delta S$$

10 $${S}_{u}={S}_{u}-\Delta S.$$

According to the above definitions of triple loss, gradient calculation, similarity matrix, degree matrix, Laplacian matrix, label propagation, and label iterative training, we introduce these components into the iterative learning process.

First, randomly initialize the network parameters *θ*, and use triple CNN train the labeled data to optimize the deep metric learning model and classification network. Second, input all the labeled samples ${S}_{l}$ and unlabeled samples ${S}_{u}$ at the same time to obtain the corresponding deep features ${S}_{l}^{^{\prime}}$ and ${S}_{u}^{^{\prime}}$, and then perform label propagation through the trained network, obtain the classification label ${L}_{u}^{1}$ and cluster label ${L}_{u}^{2}$ of the unlabeled data, compare ${L}_{u}^{1}$ and ${L}_{u}^{2}$, and repeat this iterative process continuously. Finally, use Eqs. (9–10) to iteratively update the weakly-labeled data until all unlabeled data is converted to strong-labeled data. The specific process is shown in Algorithm 2.
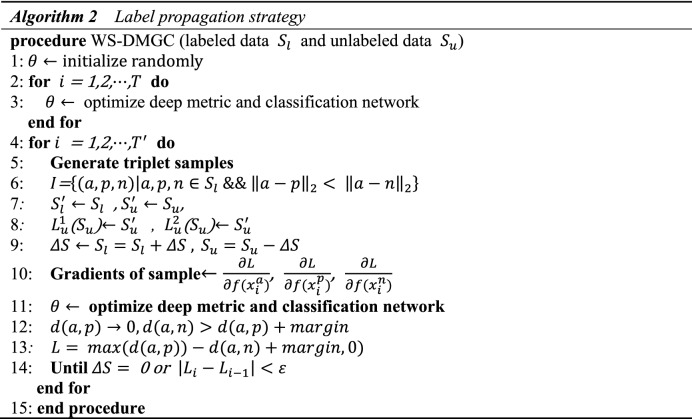


## Results

### Experimental analysis

Two image classification datasets, TCIA and Cooperative hospital, were used in this paper. Each dataset is used in a weakly supervised learning evaluation setting where part of the training images are labeled and the rest are unlabeled. Specifically, in this study, the TCIA dataset (including 1018 images) was used to train the deep network and test the hyperparameters, and the dataset from the Cooperative hospital was used for biological and clinical validation.

### TCIA

The dataset consisted of 1018 cases, 463 of which were labeled samples, including a pulmonary medical image file and corresponding XML documents for labeling and annotating lesions. Four experienced chest radiologists performed two-stage diagnostic markers on the 463 lung CT images and gave a grade 1 to 5 malignancy rating. For each case, only the image with the largest tumor area was selected for follow-up experiments. This paper uses the same method of randomly selecting marker samples as in Mean Teacher^28^. The selection process was repeated at least 10 times, using the WS-DMGC network on TCIA for different data set classification, with an average performance of more than 10 times. We follow the principle of using each marker sample, and all images have a resolution of 32 × 32. This article builds our implementation on the Pytorch code that is publicly available for the Mean Teacher^28^ method.

### Cooperative hospital

The Cooperative hospital lung cancer dataset contains CT sequence images, gene expression, DNA methylation and gene mutation data of 372 patients from five categories downloaded from the cooperative hospital. All images have a resolution of 32 × 32. We follow the same protocol as TCIA. The data set was used to demonstrate the biomedical value of this supervised classification method. In addition to genomic information, clinical data such as survival times and drug responses of patients with lung cancer and pulmonary nodules were also downloaded.

Since the goal of WS-DMGC is to improve the learning performance of the weakly supervised clustering method based on deep learning, in order to evaluate the effectiveness of the WS-DMGC method, this paper uses several existing models as the baseline of WS-DMGC and compares them with them. The original model was compared, and the experimental results were run more than 20 times on average to ensure statistical significance. include:

- Traditional unsupervised methods

FCH^13^: A PCA-based methods.

SC-CNMF^29^: A subspace clustering guided convex nonnegative matrix factorization.

- Traditional semi-supervised methods

FSLSC^17^: A feature space learning model and four FSL algorithms.

SMKL^20^: A multiple kernel learning framework for clustering and semi-supervised classification.

- Deep unsupervised methods

DFC^30^: An continuous objective function that combine the soft-partition clustering with deep embedding.

DECCA^31^: A deep embedding clustering framework based on contractive autoencoder.

- Deep semi-supervised methods

DSL^32^: A framework for data self-labeling based on deep autoencoder combined with a self-labeled technique that takes into consideration cross-entropy.

GLP^33^: A graph filtering framework-label propagation and graph convolution networks.

Shoestring^34^: An incorporates metric learning into the paradigm of graph-based semi-supervised learning.

### Dataset pre-processing

**Figure 6:**
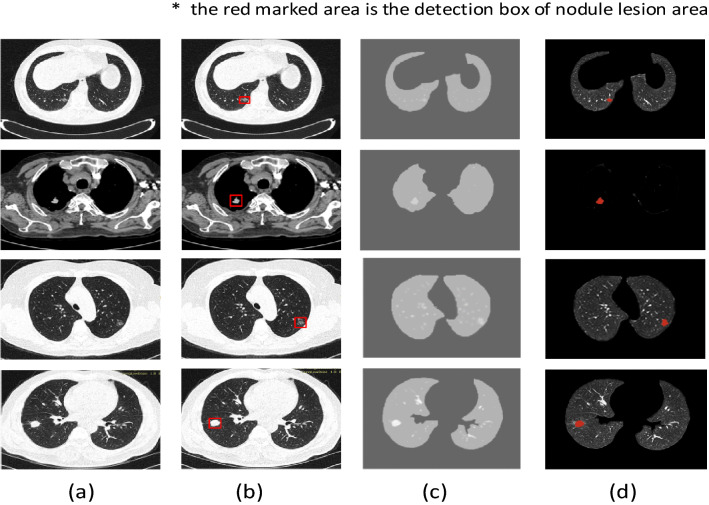
The detection and segmentation result for different kind of pulmonary nodules (**a**) normal original data (**b**) labeled data lesion area (**c**) segmentation data (**d**) multi-scale filtering enhancement data.

In terms of the detection of lesion areas, according to some previous work in our laboratory, this paper adopts an automatic detection method of lung lesions based on multi-scale enhancement filters and three-dimensional morphological features^35^. First, the adaptive threshold iteration method and the region growing algorithm are used to segment the lung parenchymal image sequence. Then, two multi-scale enhancement filters are constructed to enhance the three dimensional nodule image and blood vessel image. The 18-neighbor growth algorithm was used to extract lung nodules. Finally, detect the Region Of Interest (ROI). Figure 6 shows the detection results of different types of nodules. (a) is the original data of the cooperative hospital, (b) is the lesion area marked by the doctor, (c) is the lung parenchyma segmentation result, (d) is the detection result of multi-scale filter enhancement and 18 neighborhood growth algorithm.

### Experimental parameters

**Figure 7:**
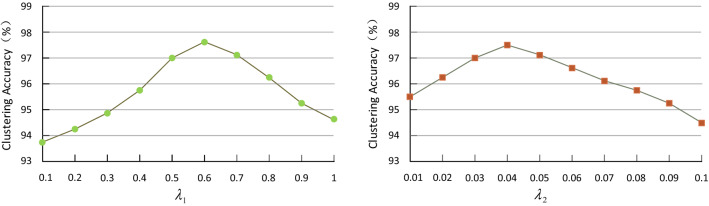
Comparison of the accuracy of different parameter values ${\lambda }_{1}$ and ${\lambda }_{2}$.

The changes of the parameters ${\lambda }_{1}$ and ${\lambda }_{2}$ in the algorithm of the WS-DMGC model mentioned above will affect the overall clustering effect. This article will adjust the parameters on the TCIA data set. For the ${\lambda }_{1}$ and ${\lambda }_{2}$ parameters, calculate the performance change of WS-DMGC in the change interval with a step length of 0.1 and a step length of 0.01, respectively. It can be seen from the results of Fig. 7 that when ${\lambda }_{1}$ is [0.5, 0.7] and ${\lambda }_{2}$ is [0.03, 0.05], WS-DMGC can achieve stable and good clustering performance. In addition, similar results can be observed on the data set of Cooperative hospitals.

### Performance metrics

The evaluation indicators of clustering results are mainly divided into internal quality evaluation standards and external quality evaluation standards. Without prior knowledge, the internal indicators only evaluate the clustering results, and use the attribute characteristics of the data set to evaluate the pros and cons of the clustering algorithm. The clustering quality is evaluated by calculating three similarities: overall similarity, average similarity between clusters or average similarity within clusters. From this, the potential distribution and internal structure of the data set samples can be found. In the case of prior knowledge, external evaluation indicators are required to compare existing tags with the output results, and finally data with the same tag are in the same cluster, and data with different tags are in different clusters.

This paper uses weakly supervised deep metric learning with prior knowledge information to guide the clustering process and obtain the results of lung cancer imaging subtype classification. Therefore, this article uses Purity, Accuracy (ACC), Normalized Mutual Information (NMI) and Silhouette Coefficient (SC)^36^ to measure and compare the performance of all algorithms. These four metrics are used to calculate the tightness within the same cluster and the dispersion between different clusters. The results show that the higher the values of Purity, ACC, NMI and SCs, the better the cluster classification effect.

Purity is a simple clustering evaluation method. It only needs to calculate the ratio of the number of correct clusters to the total number. The value range is [0,1]. The closer to 1, the better the clustering result. The definition of Purity is shown in Eq. (11):

11 $$Purity\left(\Omega ,C\right)=\frac{1}{N}\sum_{k}\underset{j}{\mathit{max}}\left|{\omega }_{k}\cap {c}_{j}\right|.$$

Among them, *N* represents the total number of samples, $\Omega = \{{\omega }_{1}, {\omega }_{2}, \cdots , {\omega }_{k}\}$ represents the cluster division, $C = \{{c}_{1}, {c}_{2}, \cdots ., {c}_{j} \}$ represents the true category division.

ACC is used to compare the label obtained by the clustering algorithm with the real label with prior information provided by the sample. The value range is [0,1]. The closer to 1 the better the clustering result. The definition of ACC is shown in Eq. (12):

12 $$ACC=\frac{{\sum }_{i=1}^{n}\delta ({s}_{i},map({r}_{i}))}{n}.$$

Among them, ${r}_{i}$ and ${s}_{i}$ respectively represent the label and real label obtained by clustering of sample ${x}_{i}$, n is the total number of samples, and *δ* represents the function as shown Eq. (13):

13 $$\delta \left(x,y\right)=\left\{\begin{array}{l}1 \; if \; x=y\\ 0, otherwise.\end{array}\right.$$

The value range of NMI is [0,1]. Let *x* and *y* be random variables for cluster assignment and class label. *I*(*x,y*) represents the mutual information between *x* and *y*, *H*(*x*) and *H*(*y*) are the entropy of *x* and *y.* NMI is defined as shown in Eq. (14):

14 $$NMI\left(X,Y\right)=\frac{I\left(X,Y\right)}{\sqrt{H\left(X\right)H\left(Y\right)}}.$$

SC is another evaluation index of clustering results, originally proposed by Peter J. Rousseeuw in 1986^37^. It combines the two factors of intra cluster and inter-cluster, which can be calculated as shown in Eqs. (15), (16) and (17):

15 $$a\left(i\right)=\frac{{\sum }_{i\in {C}_{i},i\ne {i}^{^{\prime}}}dist\left(i,{i}^{^{\prime}}\right)}{\left|{C}_{i}\right|-1},$$

16 $$b\left(i\right)=\underset{C,1\le j\le k,j=i}{\mathit{min}}\left\{\frac{{\sum }_{{i}^{^{\prime}}\in {C}_{i}}dist\left(i,{i}^{^{\prime}}\right)}{\left|{C}_{j}\right|}\right\},$$

17 $$S\left(i\right)=\frac{b\left(i\right)-a\left(i\right)}{max\left\{a\left(i\right),b\left(i\right)\right\}}.$$

The value range of SC is [− 1,1]. $a(i)$ represents the average distance from sample $i$ to other samples in the same cluster, and $\mathrm{b}(\mathrm{i})$ represents the minimum average distance from sample $i$ to all clusters that do not contain $i$. The value of $a(i)$ reflects the compactness of the cluster to which $i$ belongs. The smaller the $a(i)$, the more compact the cluster. The value of $b(i)$ captures the degree of separation of $i$ from other clusters. The larger the $b(i)$, the more separated $i$ is from other clusters. When the SC value of $i$ is close to 1, it indicates that the cluster containing $i$ is compact, and $i$ is far away from other clusters. When the value of the SC is negative, it means that the $i$ sample objects in other clusters are closer than the objects in the cluster where oneself are, and should continue training samples.

The average distance ${b}_{ij}$ from all samples $i$ to the other ${C}_{j}$ is called the difference between samples $i$ and ${C}_{j}$. The difference between the clusters of sample $i$ is defined as $b(i)=min \{{b}_{i1}, {b}_{i2}, \cdots , {b}_{ik}\}$.

## Discussions

### Performance comparison results of different loss variant networks

In order to compare the effectiveness of contrastive loss and triple loss in the label propagation algorithm, this paper defines four kinds of variant networks and compares their performance with the WS-DMGC proposed in this paper in the lung cancer subtype classification task. Details are as follows:

1. S_0_: using contrastive loss weakly-supervised deep metric learning network
2. S_1_: using triplet loss weakly-supervised deep metric learning network
3. S_2_: using label propagation network to dynamically add the labeled samples to obtain classification results
4. S_3_: using contrastive loss and label propagation strategy to classify samples
5. WS-DMGC: using triplet loss and label propagation strategy to classify samples

Table 1 shows the experimental results for different loss variant models. Using the TCIA and Cooperative Hospitals dataset, the percentage of labeled samples is set to 10%. It can be seen that the clustering performance of S1 and S2 is better than that of S0, the performance of S_3_ is sub-optimal, and the results show that using the triple loss and label propagation algorithm at the same time has the best clustering performance on both data sets.

**Table 1:** Evalutation metric on TCIA and cooperative hospitals dataset (bold indicates best).

Methods	Labeled samples	Cooperative hospitals			TCIA		
		Purity (%)	ACC (%)	NMI (%)	Purity (%)	ACC (%)	NMI (%)
S_0_	10%	85.50	86.24	86.17	86.15	91.60	90.92
S_1_	10%	86.13	88.35	89.66	88.54	92.45	91.58
S_2_	10%	86.97	90.57	90.23	89.47	95.17	94.74
S_3_	10%	87.91	91.36	91.58	89.95	96.44	96.29
WS-DMGC	10%	**89.62**	**92.00**	**93.86**	**90.10**	**97.93**	**97.85**

### Study of different label propagation schemes

In medical images, because the density of each cluster, the number of labeled samples and their distribution in each cluster are different, this paper designs a more reasonable weak supervised learning method based on graph clustering based on the k-nearest neighbors label propagation algorithm. The basic idea is to use the label information of labeled samples to dynamically predict the label information of unlabeled samples and infer the false labels of unlabeled samples. Based on this, this paper also introduces an improved graph clustering algorithm to achieve strong labeling for unlabeled samples in the iterative label propagation process. This propagation strategy is also applicable to other fields.

Table 2 lists the accuracy comparison of each semi-supervised metric learning in the TCIA dataset for different label propagation schemes. This paper considers two possible scenarios (1) no promotion, only transfer network weights (2) Nearest neighbor metric propagation. In these two cases, Siamese neural network and triple neural network are used to extract the feature representation, and the classifier is trained at the same time. We use the optimal parameters of each training method to train the model. The results show that the label propagation method in this paper will also outperform the most advanced performance in the case of few labeled samples. We achieved 95.15% accuracy by using 0.5% labeled samples. With the increase of labeled samples, the clustering results gradually tend to be optimal.

**Table 2:** Comparison of accuracy of different semi-supervised metric learning in TCIA.

Method	Propagation	0.50%	10%	20%	30%	50%	80%
SiameseNet-C	No	43.61	54.06	65.77	73.57	81.44	86.23
	Nearest neighbor	67.63	68.14	77.64	78.28	87.04	90.88
TripletNet-C	No	86.56	90.01	91.08	91.22	91.77	91.94
	Nearest neighbor	95.15	98.01	98.03	98.32	98.76	99.25

### Performance evaluation of the number of labeled samples

The paper makes experimental comparison based on two datasets of TCIA and Cooperative Hospitals, increases the percentage of labeled data from 0 to 100%, and trains the WS-DMGC network model with 20% labeled samples as the interval. To evaluate the clustering performance of the WS-DMGC method. Table 3 shows the accuracy results of the proposed method under the influence of different number of labeled samples. It can be clearly seen that the more labeled samples used in the training process, the better performance of the WS-DMGC model under the three indicators, especially in the case of all labeled samples, the clustering accuracy can reach the optimal.

**Table 3:** Comparison of performance of label propagation algorithm with different number of labeled samples.

Dataset	Metric	Number of labeled samples (%)					
		0	20	40	60	80	100
Cooperative hospitals	Purity (%)	90.56	99.08	99.14	99.16	98.67	99.05
	ACC (%)	88.07	98.99	99.12	98.95	99.10	99.25
	NMI (%)	76.42	93.73	94.56	95.27	96.73	97.02
TCIA	Purity (%)	76.45	90.98	91.35	93.21	95.68	96.94
	ACC (%)	89.71	98.45	98.46	98.92	98.76	98.92
	NMI (%)	90.33	97.89	98.01	98.54	99.06	99.34

However, in the field of medical imaging, due to the difficulty of image annotation and the high cost of annotation, image annotations are often difficult to obtain, which is a practical problem in the development of medical imaging research. Therefore, this paper wants to solve the dependence of deep models on labeled samples to the greatest extent, especially in the case of a small number of labeled samples, if the optimal performance can be achieved, It will greatly highlight the advantages of the proposed model, and train a more general weakly supervised learning algorithm while reducing the cost of sample labeling.

### Clustering performance evaluation

In order to evaluate the effectiveness of the model proposed in this paper, the deep weakly supervised learning method in this paper also compares different indicators with traditional unsupervised learning methods, traditional semi-supervised learning methods, deep unsupervised learning methods and deep semi-supervised learning methods on two data sets. Experiments were carried out on the different percentages of labeled samples, and the experimental results are shown in Tables 4 and 5.

**Table 4:** Comparison results in terms of three metrics (purity, ACC and NMI) on TCIA dataset.

	Methods	Percentages					
		Metric	0.50%	1%	2%	5%	10%
Traditional unsupervised methods	FCH	Purity (%)	54.35	55.42	56.24	58.98	64.25
		ACC (%)	65.74	64.26	67.68	67.93	69.45
		NMI (%)	59.45	59.86	62.13	65.26	67.13
	SC-CNMF	Purity (%)	61.21	67.31	62.13	70.59	71.25
		ACC (%)	70.84	71.25	71.76	72.34	74.85
		NMI (%)	69.60	70.06	71.38	72.49	76.67
Traditional semi-supervised methods	FSLSC	Purity (%)	59.35	67.21	64.78	72.16	74.23
		ACC (%)	74.26	74.59	74.91	75.76	78.32
		NMI (%)	86.24	86.71	87.35	88.64	90.26
	SMKL	Purity (%)	60.43	62.34	67.98	71.12	73.45
		ACC (%)	75.74	76.26	76.89	76.93	77.68
		NMI (%)	85.33	85.57	86.84	86.93	87.65
Deep unsupervised method	DFC	Purity (%)	72.91	71.35	73.32	74.28	75.13
		ACC (%)	72.93	73.54	74.21	75.55	78.39
		NMI (%)	80.23	80.68	81.34	82.69	84.17
	DECCA	Purity (%)	73.63	74.44	74.49	75.13	76.42
		ACC (%)	84.06	84.69	85.71	87.49	90.43
		NMI (%)	80.24	80.79	81.35	82.64	83.25
Deep semi-supervised method	DSL	Purity (%)	77.28	78.32	78.97	79.58	80.79
		ACC (%)	83.54	83.85	84.56	86.17	89.48
		NMI (%)	85.46	85.87	86.05	86.73	87.24
	GLP	Purity (%)	81.39	82.13	82.32	83.63	84.11
		ACC (%)	95.13	**97.12**	97.35	97.64	97.97
		NMI (%)	84.43	85.07	85.94	86.53	88.45
	Shoestring	Purity (%)	81.11	81.23	84.01	84.89	86.55
		ACC (%)	86.14	87.56	88.43	89.64	92.36
		NMI (%)	**95.25**	**95.64**	95.93	96.35	97.68
	WS-DMGC	Purity (%)	**83.23**	**83.98**	**84.35**	**85.44**	**90.15**
		ACC (%)	**95.15**	95.96	**97.48**	**97.77**	**98.01**
		NMI (%)	95.12	95.17	**96.58**	**96.89**	**97.85**

**Table 5:** Comparison results in terms of three metrics (purity, ACC and NMI) on cooperative hospitals dataset.

	Methods	Percentages					
		Metric	0.50%	1%	2%	5%	10%
Traditional unsupervised methods	FCH	Purity (%)	60.45	56.87	57.05	68.69	73.19
		ACC (%)	65.59	72.09	72.29	75.23	76.24
		NMI (%)	60.17	67.66	67.71	71.25	71.77
	SC-CNMF	Purity (%)	64.53	74.08	74.52	77.89	81.57
		ACC (%)	72.33	74.18	75.65	77.64	78.44
		NMI (%)	64.56	65.89	68.32	68.78	74.03
Traditional semi-supervised methods	FSLSC	Purity (%)	72.16	74.25	77.46	81.44	82.00
		ACC (%)	74.40	74.45	76.59	76.94	80.47
		NMI (%)	71.23	72.14	72.23	73.23	77.32
	SMKL	Purity (%)	70.80	75.69	77.40	81.33	81.76
		ACC (%)	79.55	79.57	79.79	79.91	80.74
		NMI (%)	71.58	74.20	74.96	76.04	78.03
Deep unsupervised method	DFC	Purity (%)	71.64	74.63	76.76	80.23	80.55
		ACC (%)	75.29	76.11	76.32	76.41	77.91
		NMI (%)	74.56	75.16	75.16	75.68	77.83
	DECCA	Purity (%)	76.52	77.31	80.12	91.64	84.99
		ACC (%)	80.33	80.67	80.90	80.97	81.61
		NMI (%)	76.39	80.03	80.26	81.68	81.77
Deep semi-supervised method	DSL	Purity (%)	91.54	93.01	93.46	94.56	95.84
		ACC (%)	92.97	94.29	94.33	95.21	95.57
		NMI (%)	79.25	86.29	87.53	90.65	90.87
	GLP	Purity (%)	91.46	94.60	95.32	95.42	96.92
		ACC (%)	92.73	93.46	94.15	94.29	96.67
		NMI (%)	79.62	95.91	87.45	87.97	90.83
	Shoestring	Purity (%)	92.88	94.25	95.10	95.24	95.89
		ACC (%)	92.04	93.76	93.78	94.01	95.44
		NMI (%)	78.09	86.20	89.74	90.67	91.26
	WS-DMGC	Purity (%)	96.32	98.00	98.25	98.67	99.03
		ACC (%)	93.85	97.11	98.46	98.68	98.96
		NMI (%)	80.32	88.26	91.56	92.15	92.84

Tables 4 and 5 show the clustering results of TCIA dataset and cooperative hospital dataset. According to the experimental results, the WS-DMGC method proposed in this paper is better than the most advanced methods when the percentage of labeled samples is 10%, and has better classification performance than most methods.

In addition, we specifically evaluated the effectiveness of the proposed weakly supervised deep metric learning algorithm by comparing it with metric learning methods in two literatures, including : (1)SCDML^38^; (2) SCDMLGE^19^.

As shown in Table 6, on the widely used TCIA dataset, our model achieves a new record accuracy of 95.15%. On the Shanxi Provincial Cooperative Hospital dataset, it reaches 93.85%. More importantly, we can achieve state-of-the-art lung cancer imaging subtype recognition performance using only a small amount of labeled data.

**Table 6:** Performance comparison of different algorithms on two datasets based on Purity, ACC and NMI.

Datasets	Evaluation (%)	SCDML^30,38^	SCDMLGE^19^	WS-DMGC
Cooperative hospitals	Purity	90.37	95.64	96.32
	ACC	88.32	93.21	93.85
	NMI	74.96	79.05	80.32
Labeled data		0.50%	0.50%	0.50%
TCIA	Purity	77.31	82.15	83.23
	ACC	89.29	94.29	95.15
	NMI	89.74	93.88	95.12
Labeled data		0.50%	0.50%	0.50%

### Selection of cluster quantity

**Figure 8:**
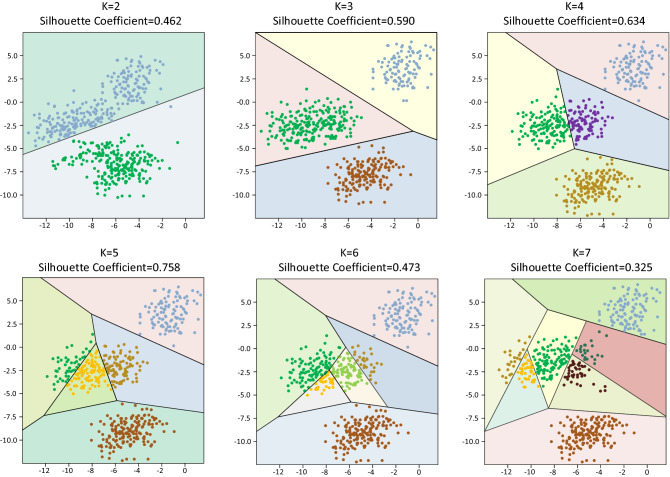
Silhouette coefficients analysis of different clustering categories.

According to the principle of “dense inside and sparse outside”, that is, the samples within the same cluster should be dense enough, and the samples between different clusters should be sufficiently distant. In this paper, the SC method is used to select the optimal value of the number of clustering categories. The contour plot shows the distance between each point in a cluster and the points in adjacent clusters. The range of this measurement is [− 1,1]. The closer to 1 indicates that the number of clusters makes the classification effect of the sample better, 0 indicates that it is between the two, and the closer to − 1 indicates that the cluster where the sample is located is wrong. When the percentage of labeled samples of WS-DMGC is set to 10%, and the parameter value ${\lambda }_{1}$ is 0.6 and ${\lambda }_{2}$ is 0.04, the contour coefficient analysis of different cluster categories is shown in Fig. 8.

**Figure 9:**
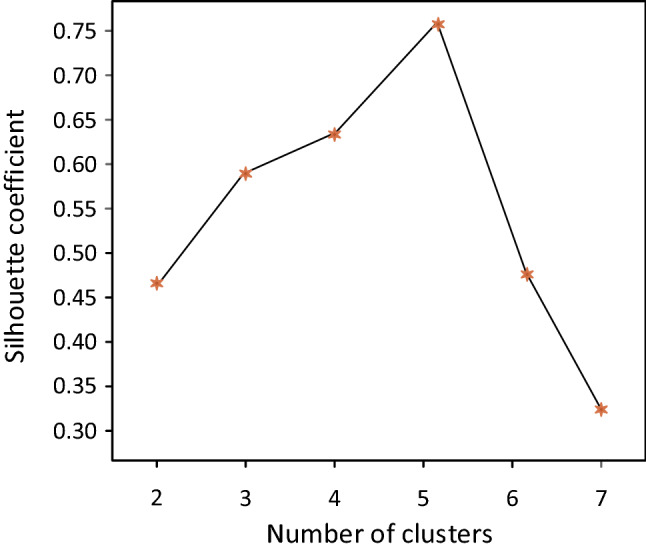
The relation curve between the Silhouette coefficient and the number of different kinds of clusters.

It can be seen from the Silhouette coefficients in Fig. 8 and the relationship curve with Fig. 9 that for a given data, the average Silhouette coefficients is the highest when the cluster category is 5 clusters, 2, 3, 4, 6, 7 clusters the time-average profile coefficients are all lower than 5 clusters. Therefore, it is optimal to divide into 5 clusters, which matches the data set with prior knowledge information.

### Evaluation of imaging subtypes of different lung cancer patients

#### Survival analysis

**Figure 10:**
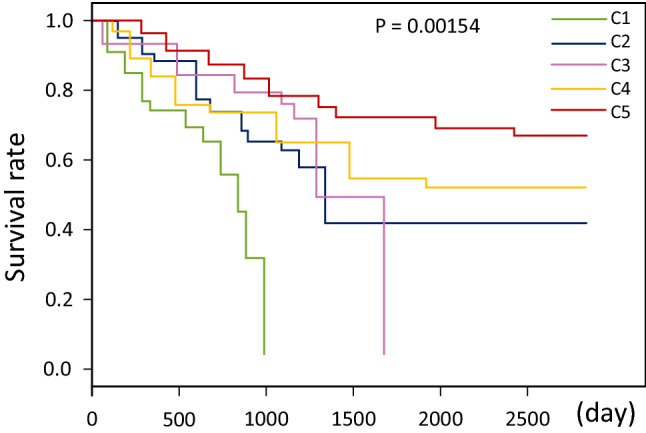
Kaplan–Meier survival curves for five lung cancer subtypes.

In order to further prove the biological significance and clinical significance of the classification results in this paper, the survival analysis of the method proposed in this paper was carried out according to the study of Long et al.^39,40^. Table 7 shows the survival time analysis of the five lung cancer imaging subtypes classified by the WS-DMGC model in the cooperative hospital data set in this paper. Figure 10 is a Kaplan–Meier diagram of five lung cancer imaging subtypes. It can be seen that the survival rate between each type of patient is very different, especially the red curve/cluster seems to have a better survival rate or prognosis than the other clusters. Kaplan–Meier curve showed significant differences in survival rates of different subtypes (log-rank, p < 0.00154).

**Table 7:** Survival time of different subtypes of lung cancer.

	C1	C2	C3	C4	C5
Mean survival time (days)	983.15	1346.32	1692.21	1954.97	2465.65
900 day survival rate	69.4%	71.1%	79.3%	75.6%	82.9%
1500 day survival rate	32.3%	42.9%	50.3%	54.8%	71.5%

It can be seen from Table 7 and Fig. 10 that the survival rate of patients gradually increases from Class 1 to Class 5. The life expectancy of patients with type 1 and 2 cancers is 10–40% shorter than that of the other three groups. In category 3, category 4, and category 5 patients, more than 75% of patients survived for more than 900 days. More than 50% of cancer patients live more than 1500 days, and in the 5 categories, this proportion rises to 71.5%. These results show that the clustering WS-DMGC model in this paper can use CT image data to distinguish the clinical differences of lung cancer imaging subtypes.

### Biological analysis

**Figure 11:**
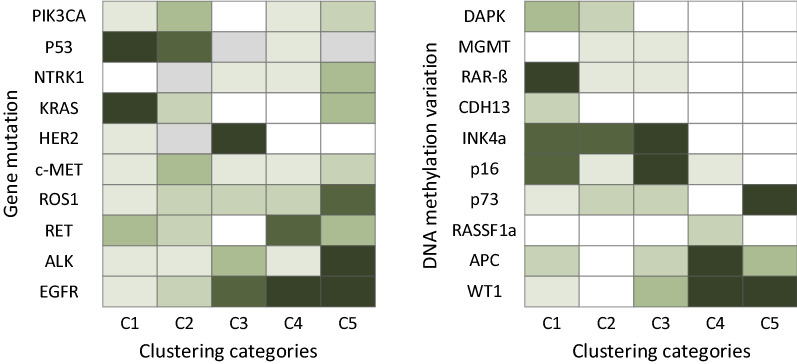
Lung cancer (partial) gene mutation and DNA methylation variation frequency map for every subtype.

In order to further analyze the relationship between lung cancer subtypes and their specific differences at the biomolecular level, this paper selected the top 10 mutations^41,42^ with the largest positive correlation with each lung cancer imaging subtype, and based on statistics, gene mutation^43^ and DNA methylation variation profile analysis for each subtype. The correlation map of each type of gene mutation and DNA methylation variation is shown in Fig. 11. The darker the color, the higher the frequency of mutation.

It can be seen from Figs. 10 and 11 that the average survival time of C4 and C5 is much higher than that of other categories. The mutation frequency of EGFR and ALK is particularly prominent in these two groups of people, and there are basically no mutations in other groups. This mutation indicates that EGFR and ALK genes are characteristic mutations of C4 and C5 subtypes. Interestingly, samples with EGFR and ALK gene mutations are divided into clusters 4 and 5. However, C4 includes all those with APC samples of methylation mutations. C3 has a higher mutation rate for HER2 and EGFR genes. C1 and C2 are more pronounced in KRAS and P53 gene mutations, but the degree of genetic mutation of RET is not the same in these two types of subtypes, and the mutation of RAR-$\upbeta$ methylation is in these two types of subtypes there are also significant differences. The frequency of P53 gene mutations gradually decreases from C1 to C5, indicating that the corresponding survival time of patients will be longer and longer, indicating that P53 gene mutations will lead to a decrease in survival rate. As the disease worsens, the types of mutant genes are also undergoing significant changes. These results show that the network architecture of this paper can also accurately capture the expression of DNA methylation on lung cancer subtypes without using DNA methylation data. This shows that the new weakly supervised classification model proposed in this paper can identify subtypes that reflect disease mechanisms at the molecular level.

## Conclusion

This paper proposes a weakly supervised learning model based on deep metric learning and graph clustering, which is used to learn clinical situations where there are only a few labeled samples. This paper proposed a new framework WS-DMGC based on traditional metric learning and clustering assumptions. Use the method of embedding the online triple neural network into the deep metric learning network to form a new label propagation network. The WS-DMGC network using triple loss function can extract more powerful discriminative features, and then learn more discriminative indicators. The label propagation network is used to alternate iteratively update the unlabeled data so that it becomes clinically usable labeled data, thereby improving the accuracy of network clustering and providing more accurate subtype categories for the clinic.

The proposed method was verified on the TCIA dataset and the Shanxi Provincial Cooperative Hospital dataset, which proved the effectiveness and robustness of this method for the classification of lung cancer imaging subtypes. Survival analysis and biological analysis were performed to verify the imaging subtypes of different lung cancer patients. Kaplan–Meier survival curve was used to evaluate whether the subtype has medical value. Correlation analysis was used to find the differentially expressed genes and mutated genes associated with each cluster. The specific gene variation of each type led to the change of molecular pathway, and the change of molecular pathway of lung cancer gene was the key basis for targeted treatment. Finally, by exploring the potential biological mechanism of imaging subtypes, it can provide a reference for doctors to design targeted treatment programs. In the future work, we will try to improve the availability of a small amount of clinically acquired labeled data through more data enhancement methods.

## Data Availability

The data that support the findings of this study are available from Department of Radiology of Shanxi Province Cancer Hospital, but restrictions apply to the availability of these data, which were used under license for the current study, and so are not publicly available. Data are however available from the authors upon reasonable request and with permission of Department of Radiology of Shanxi Province Cancer Hospital. If anyone would like data from this study, please contact the authors at renxueting0085@link.tyut.edu.cn.
